# Disruption of Cell Membranes and Redox Homeostasis as an Antibacterial Mechanism of Dielectric Barrier Discharge Plasma against *Fusarium oxysporum*

**DOI:** 10.3390/ijms25147875

**Published:** 2024-07-18

**Authors:** Shiqian Yu, Jiajin Sun, Haiming Chen, Weijun Chen, Qiuping Zhong, Ming Zhang, Jianfei Pei, Rongrong He, Wenxue Chen

**Affiliations:** College of Food Sciences & Engineering, Hainan University, 58 People Road, Haikou 570228, China

**Keywords:** direct barrier discharge plasma, *Fusarium oxysporum*, antibacterial mechanism, free fatty acid metabolism

## Abstract

Direct barrier discharge (DBD) plasma is a potential antibacterial strategy for controlling *Fusarium oxysporum* (*F. oxysporum*) in the food industry. The aim of this study was to investigate the inhibitory effect and mechanism of action of DBD plasma on *F. oxysporum*. The result of the antibacterial effect curve shows that DBD plasma has a good inactivation effect on *F. oxysporum*. The DBD plasma treatment severely disrupted the cell membrane structure and resulted in the leakage of intracellular components. In addition, flow cytometry was used to observe intracellular reactive oxygen species (ROS) levels and mitochondrial membrane potential, and it was found that, after plasma treatment, intracellular ROS accumulation and mitochondrial damage were accompanied by a decrease in antioxidant enzyme activity. The results of free fatty acid metabolism indicate that the saturated fatty acid content increased and unsaturated fatty acid content decreased. Overall, the DBD plasma treatment led to the oxidation of unsaturated fatty acids, which altered the cell membrane fatty acid content, thereby inducing cell membrane damage. Meanwhile, DBD plasma-induced ROS penetrated the cell membrane and accumulated intracellularly, leading to the collapse of the antioxidant system and ultimately causing cell death. This study reveals the bactericidal effect and mechanism of the DBD treatment on *F. oxysporum*, which provides a possible strategy for the control of *F. oxysporum*.

## 1. Introduction

Food spoilage caused by fungi is a non-negligible problem in the food industry. The presence of fungi in food products results in visible mycelium and off-flavors, which adversely affect the organoleptic value of food. Unfortunately, fungi can also produce potential carcinogenic toxin metabolites, thereby compromising the safety of food [1]. *Fusarium* is a complex group of pathogenic fungi that has become one of the most important pathogenic fungi in the world. *Fusarium* can cause a variety of diseases, such as root rot, canker, and blight at any stage of fruit and vegetable growth [2]. Currently, chemical agents are often used to control *Fusarium oxysporum* (*F. oxysporum*). It was found that monoterpene phenol thymol (2-isopropyl-5-methylphenol) has effective antifungal effects on *F. oxysporum* both in vitro and in vivo [3]. It has been reported that chitosan significantly inhibited the mycelial growth, spore germination, and pathogenicity of *F. oxysporum* [4]. In addition, Li et al. [5] found that the combination of phytohormone salicylic acid and rapamycin inhibited the growth of *F. oxysporum*. However, chemical agents may adversely affect the quality of food while inhibiting *F. oxysporum*.

In recent years, more and more non-thermal sterilization techniques are being used in the processing of various types of food products [6], such as high-pressure processing (HPP), ultrasonic techniques, pulsed electric field (PEF), and non-thermal plasma [7]. Among them, plasma sterilization technology is widely applied in food processing because of its convenient operation and obvious sterilization effect. Plasma is an ionized gas containing a large number of reactive oxygen species (ROS), reactive nitrogen species (RNS) radicals, and charged particles [8]. Cold plasma sterilization causes the death of microorganisms mainly by ionizing the working gas to produce biologically active substances, such as ozone, charged particles, and oxygen radicals [9,10]. Many studies have shown that the use of plasma sterilization in the food industry is effective in inactivating bacteria, yeasts, and molds in food products [11]. In a previous study on the non-thermal plasma inhibition of the growth and aflatoxin production of *Aspergillus flavus*, it was shown that the accumulation of plasma-generated ROS could destroy the mitochondria of *Aspergillus flavus* spores and induce fungal apoptosis. In addition, by regulating the expression of genes related to the aflatoxin biosynthesis pathway, the production of aflatoxin B_1_ (AFB_1_) can be significantly inhibited [12]. Another study showed that ^1^O_2_ produced by plasma can induce mitochondrial depolarization and protein oxidation/aggregation, promoting cell apoptosis [13]. However, the biological effects of the plasma treatment on fungal spores and the mechanism behind the antibacterial effects are still poorly understood.

In this study, we investigated the inhibition mechanism of a direct barrier discharge (DBD) plasma treatment on *F. oxysporum* in mango juice. We observed the morphology of plasma-treated spores under field emission transmission electron microscopy and investigated the effect of plasma on the integrity of the cell membrane in conjunction with the leakage of intracellular components. For this study, flow cytometry was used for the determination of intracellular ROS levels, and in addition, we examined ROS-related enzyme levels and mitochondrial membrane potential to study the effect of plasma on the intracellular antioxidant reduction system. Finally, we explored the inhibitory mechanism of the DBD treatment on *F. oxysporum* combined with the changes in free fatty acid content. This study provides a new potential sterilization strategy for *F. oxysporum*.

## 2. Results and Discussion

### 2.1. Bactericidal Effect of DBD on F. oxysporum

**Figure 1:**
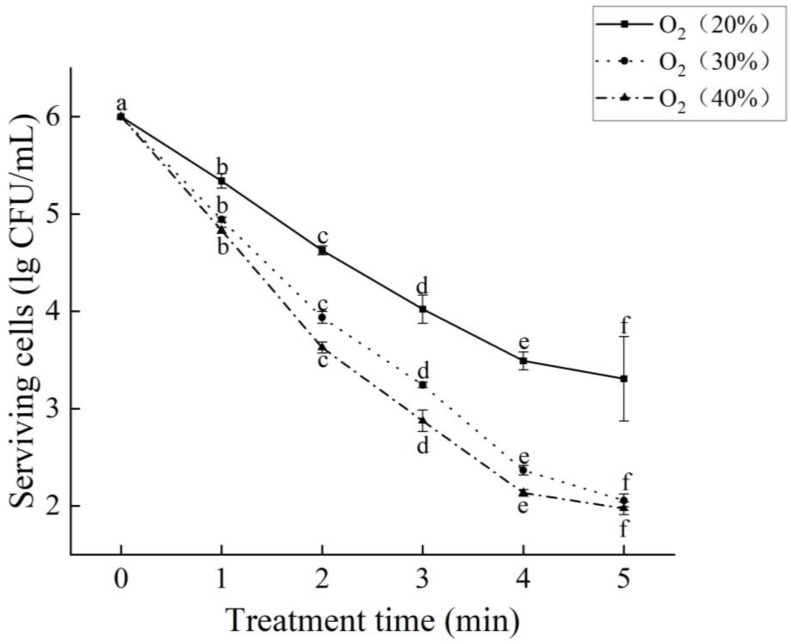
Bactericidal effect of DBD on *F. oxysporum*. The data marked with different lowercase letters in the same group indicates significant difference (*p* < 0.05).

As shown in Figure 1, the number of surviving spores gradually decreased as the treatment time increased from 1 to 5 min, consistent with the findings of Hu et al. [14], in which prolonging the DBD plasma treatment time improved the sterilization efficiency. Compared to the untreated group, the number of surviving spores decreased by 2.7, 3.95, and 4.03 log CFU/mL after the 5 min treatment of samples with an O_2_ content of 20%, 30%, and 40%, respectively. A similar study utilizing DBD plasma to treat *E. coli* and *Bacillus subtilis* also found that increasing the treatment time significantly enhanced the bactericidal efficiency [15]. After increasing the O_2_ content from 20% to 40% and treating with DBD plasma for 5 min, the number of surviving spores decreased by 1.33 log CFU/mL. The data obtained support the idea that increasing the O_2_ content of the gas led to a gradual decrease in the number of viable spores when treated with DBD plasma for the same amount of time. It has been shown that the choice of the type of working gas determines the type and amount of reactive material produced by the plasma [16,17]. A previous study using plasma to treat *Escherichia coli* and *Listeria monocytogenes* also found that the treatment with a working gas with a high O_2_ content significantly reduced the viability of the strains [18]. The possible explanation for this is that the addition of O_2_ helps to produce more ROS. ROS plays an important role as a signaling molecule in a variety of cell biological pathways, and ROS is known to induce intracellular oxidative stress and programmed cell death [19,20].

### 2.2. Morphological Changes Induced in Fungal Spores by DBD

**Figure 2:**
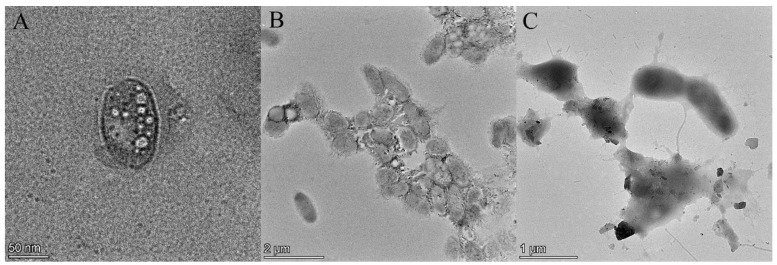
Field emission transmission electron microscopy. (**A**) Not treated with DBD plasma; (**B**) DBD plasma was applied for 3 min; and (**C**) DBD plasma was applied for 5 min.

The FETEM images of *F. oxysporum* spores treated with DBD plasma are shown in Figure 2A–C. The cell membrane and cell wall of the untreated *F. oxysporum* spores were morphologically intact and structurally clear; in addition, there was no peripheral exudation of cell contents (Figure 2A). In contrast, spores treated with DBD plasma for 3 min showed cell membrane indentation and cell membrane rupture in some spores (Figure 2B). When treated for 5 min, a large amount of exudation of cellular contents was observed (Figure 2C). The results are similar to those of previous studies, in which fungal spores also showed significant damage and aggregation after plasma treatment [21,22,23]. Another study also revealed that the non-thermal plasma treatment resulted in significant changes in the morphology of *F. oxysporum* accompanied by the loss of plasma membrane integrity [23]. Changes in spore morphology may be due to the rupture of the cell membrane, leading to the exudation of material from the spore [24]. More importantly, ROS produced by DBD plasma can cause oxidative damage to cell membranes, especially the membrane lipids near the cell surface, which contain polyunsaturated fatty acids and are sensitive to ROS [25,26,27]. Previous studies on the treatment of *E. coli* with floating electrode dielectric blocking discharge (FE-DBD) found that plasma-generated ROS induced lipid peroxidation in *E. coli* membranes, leading to membrane damage [26,28]. In addition, it has been shown that cell membranes were also damaged by electrostatic forces generated by the aggregation of charged particles produced by plasma [29].

### 2.3. Effect of DBD Plasma on Membrane Integrity of Fungal Spores

**Figure 3:**
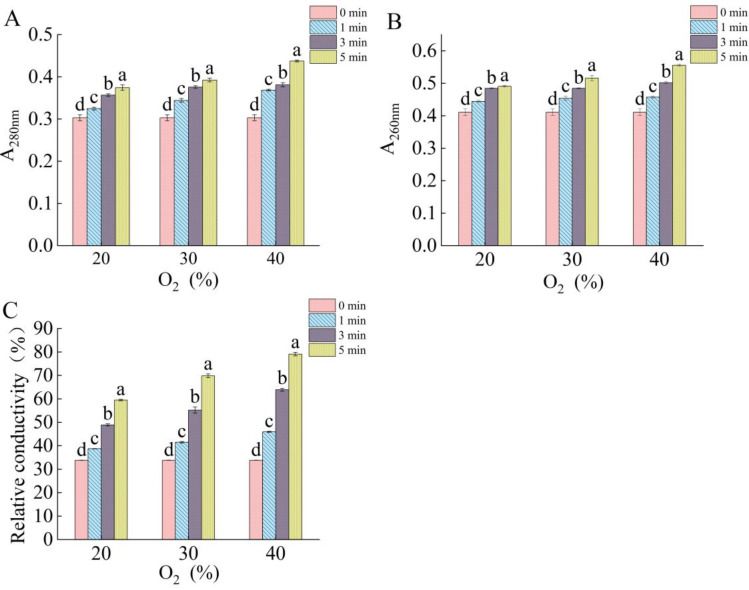
Effect of the DBD plasma treatment on the cell content leakage. (**A**) Protein leakage; (**B**) nucleic acid leakage; and (**C**) relative electrical conductivity. The data marked with different lowercase letters in the same group indicates significant difference (*p* < 0.05).

The cell membrane is an important barrier that protects the cell. In this study, we determined the cell membrane integrity according to the protein leakage, nucleic acid leakage, and relative conductivity. Figure 3A–C show the leakage of proteins and nucleic acids and changes in the relative conductivity of *F. oxysporum* after the DBD plasma treatment. With the extension of the treatment time and the increase in the O_2_ content, the leakage of proteins and nucleic acids increased as well as the conductivity. When the treatment concentration of O_2_ was increased from 20% to 40%, while time was fixed at 5 min, the leakage of proteins and nucleic acids and the relative conductivity increased by 16.83%, 13.17%, and 33%, respectively. At the same O_2_ content, the leakage of proteins and nucleic acids and the relative conductivity increased with the extension of the treatment time. When the O_2_ content was 40%, the protein and nucleic acid leakage and the relative conductivity increased by 18.73%, 21.5%, and 72.23%, respectively, for the 5 min compared to the 1 min treatment. Protein and nucleic acid leakage, as well as the relative conductivity, were at their maximum levels at 40% oxygen for 5 min. The results of the leakage of the intracellular components are in agreement with the results observed by FETEM, in which the DBD plasma treatment led to the disruption of the spore structure. The bombardment of spores by the reactants in the plasma produces an etching effect on the cell surface. This etching action erodes the cellular material, such as chitin and lactone amylase on the cell membrane. As a result, the cell membrane ruptures and the contents leak out, eventually leading to bacterial death [14].

### 2.4. Effects of DBD Plasma on Intraspore ROS Levels

**Figure 4:**
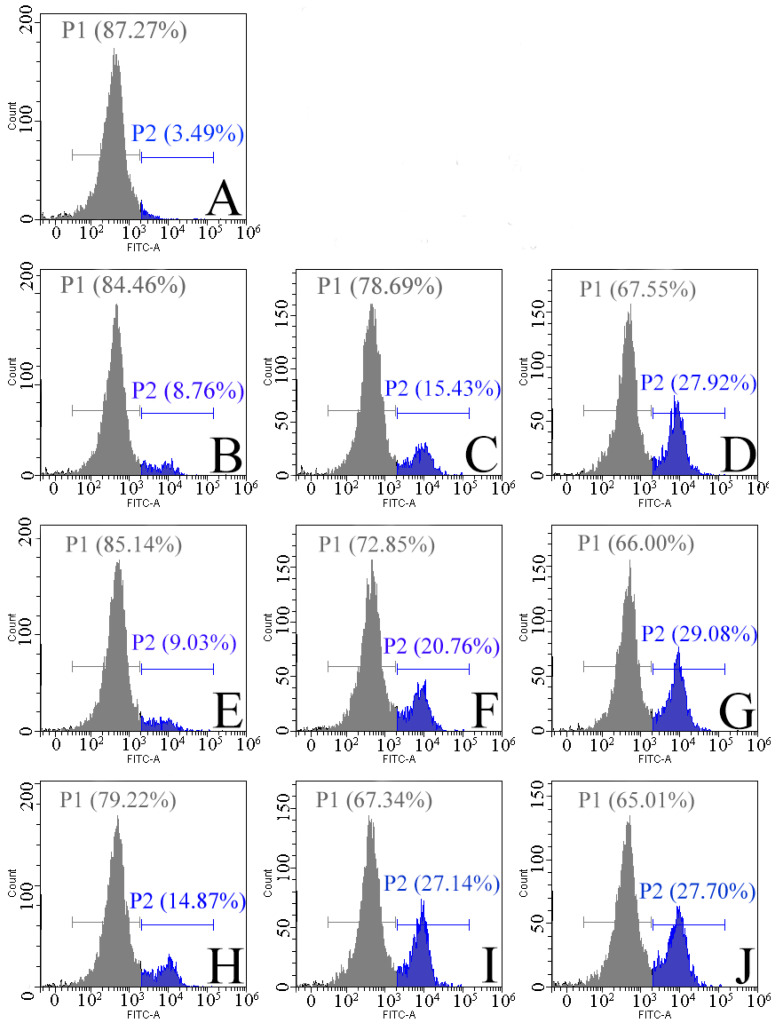
Intracellular ROS levels in *F. oxysporum* after the DBD treatment. Untreated (**A**); O_2_ content of 20%, with treatment for 1 min (**B**), 3 min (**C**), and 5 min (**D**); O_2_ content of 30%, with treatment for 1 min (**E**), 3 min (**F**), and 5 min (**G**); O_2_ content of 40%, with treatment for 1 min (**H**), 3 min (**I**), and 5 min (**J**).

Oxidative stress induced by ROS may be an important cause of spore structural damage and intracellular component leakage. Therefore, a fluorescent probe (2′,7′-dichlorodihydrofluorescein) was used to detect intracellular ROS levels in the next experiment. 2′,7′-dichlorodihydrofluorescein (DCFH-DA) itself is non-fluorescent and can freely cross the cell membrane. After entering the cell, it can be hydrolyzed by the esterase enzymes widely present in the cell to generate dichlorodihydrofluorescein diacetate (DCFH), which is not cell-membrane-permeable and thus is blocked in the cell. Non-fluorescent DCFH can be oxidized by intracellular ROS to produce the fluorescent 2′,7′-dichlorofluorescein (DCF), which generates a fluorescent signal and whose intensity is positively correlated with the intracellular ROS level [30]. In this study, we used flow cytometry to detect intracellular ROS levels. The results are shown in Figure 4, with cell cluster 1 indicating the ROS-negative cell population and cell cluster 2 indicating the ROS-positive cell population. Increasing both the treatment time and the O_2_ content of the working gas led to an increase in the number of positive cells compared to the untreated group, indicating an elevated level of intracellular ROS content. When the O_2_ content was 40%, the proportion of positive cells reached 29.7% after the 5 min treatment. An excessive intracellular ROS content can have serious consequences for the cell; therefore, ROS in the cell is usually controlled at a low level by the redox system [31,32]. In this study, the data showed that the intracellular ROS levels exceeded normal levels, possibly because ROS disrupted the permeability of the cell membrane and entered the cell and accumulated there [33]. The accumulation of ROS can disrupt the cellular antioxidant system, induce oxidative stress, and lead to cell death [34,35].

### 2.5. Effects of DBD Plasma on Contents of Redox System-Related Components

**Figure 5:**
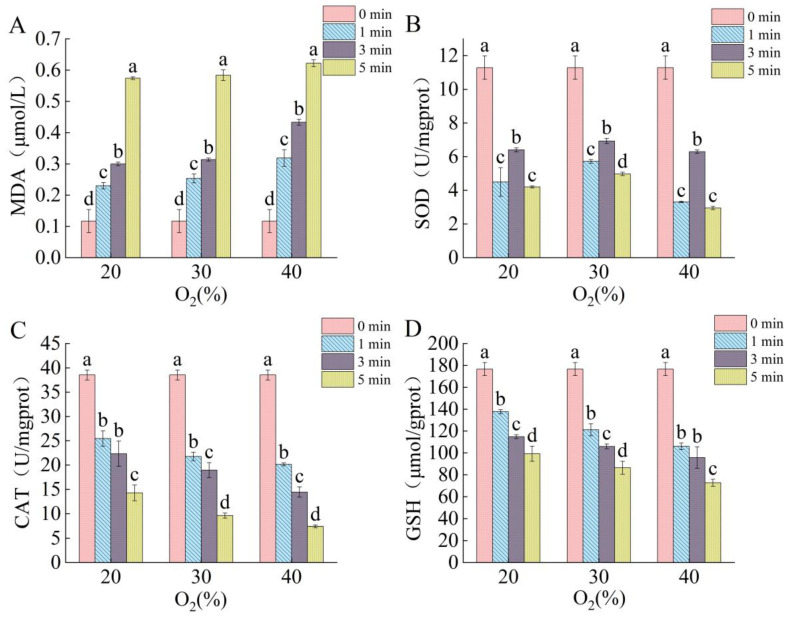
Changes in redox system-related components. (**A**) The content of MDA in *F. oxysporum* after treatment with DBD plasma. The activity of SOD (**B**) and CAT (**C**) in *F. oxysporum* after treatment with DBD plasma. (**D**) The content of GSH in *F. oxysporum* after treatment with DBD plasma. The data marked with different lowercase letters in the same group indicates significant difference (*p* < 0.05).

Lipid membranes are one of the primary targets of bioactive substances produced by plasma, and after the plasma treatment, lipids are severely oxidized by bioactive substances produced by plasma, such as ROS [34]. As shown in Figure 5A, the malondialdehyde (MDA) content increased with the increase in the plasma treatment time and O_2_ content in the working gas. When the O_2_ content was 40%, the MDA content reached 0.622 μmol/L after the DBD plasma treatment for 5 min, which is 1.7 times higher than the MDA content after the 1 min treatment with 20% O_2_. It can be seen that increasing the plasma treatment intensity increases the degree of lipid peroxidation. Membrane lipid peroxidation often results in damage to the cell membrane and the leakage of the cell contents [26]. Thus, ROS-induced lipid peroxidation may be an important factor in the aforementioned membrane damage and leakage of intracellular components.

The regulation of the intracellular antioxidant enzyme system is essential for maintaining intracellular redox homeostasis [36]. When the cell membrane is damaged, exogenous ROS enter the cell and accumulate intracellularly, which in turn affects the intracellular redox system. Antioxidant enzymes (SOD and CAT) and antioxidants (GSH) are important components of the redox system [37]. SOD catalyzes the formation of molecular O_2_ and H_2_O_2_ from $O_{2}^{-}$. CAT further catalyzes the formation of H_2_O from H_2_O_2_. GSH is also important in the cellular antioxidant system by reacting with ROS [32,33,38]. Therefore, in order to further investigate the response mechanism of the antioxidant system under oxidative stress, it is necessary to evaluate the effect of the plasma treatment on CAT and SOD activity and GSH content in spores [39]. As shown in Figure 5, the SOD activity increased after 3 min of the DBD treatment, indicating that the cells tried to eliminate the imbalance of the intracellular oxidation level caused by the invasion of extracellular oxidizing species. With the extension of the treatment time and the increase in the O_2_ content in the working gas, the SOD and CAT activity and GSH content decreased continuously. When the oxygen content was 40% and the treatment lasted for 5 min, the SOD and CAT activity as well as the GSH content decreased by 80.7%, 73.87%, and 58.83%, respectively, compared to the untreated group. The significant decrease in the antioxidant enzyme activity indicates that the cellular antioxidant system was seriously damaged [40]. Studies have shown that ROS may reduce enzyme activity by destroying the active center of the enzyme [33,41,42]. Therefore, based on the obtained data, we believe that the accumulation of ROS leads to a decrease in the activity of antioxidant enzymes, which destroys the cellular antioxidant system and ultimately causes cell death.

### 2.6. Effect of DBD Plasma on Mitochondrial Membrane Potential

**Figure 6:**
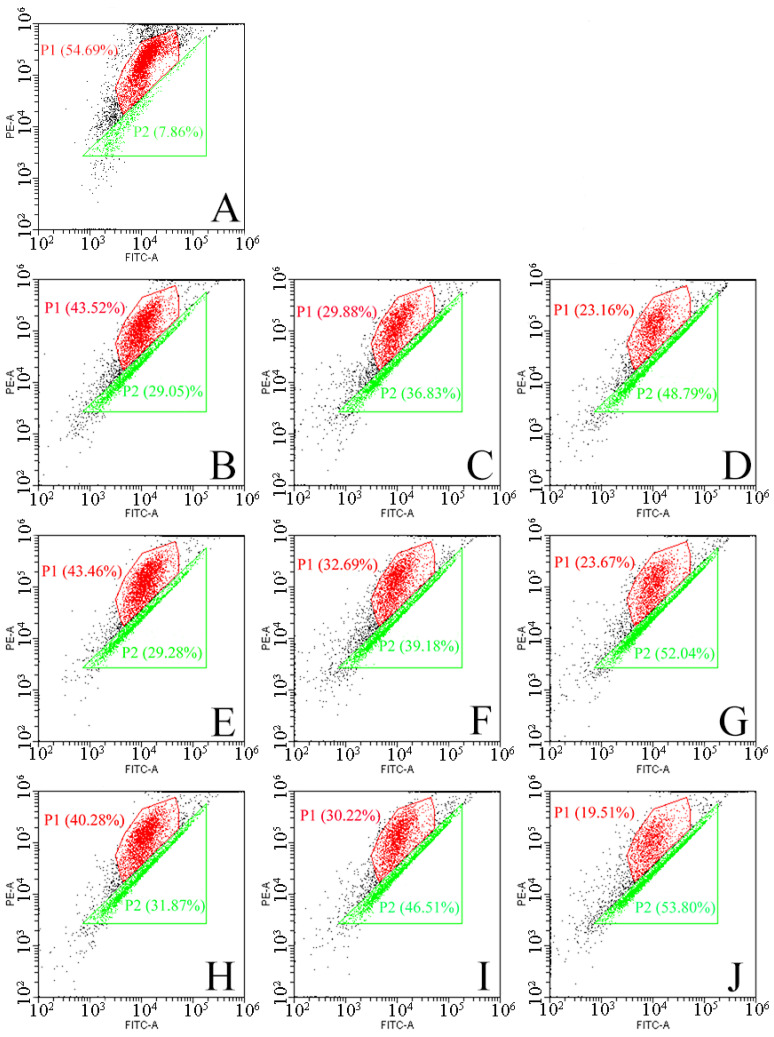
Effect of the DBD treatment on the mitochondrial membrane potential of *F. oxysporum*. Untreated (**A**); O_2_ content of 20%, with treatment for 1 min (**B**), 3 min (**C**), and 5 min (**D**); O_2_ content of 30%, with treatment for 1 min (**E**), 3 min (**F**), and 5 min (**G**); O_2_ content of 40%, with treatment for 1 min (**H**), 3 min (**I**), and 5 min (**J**).

In eukaryotic cells, mitochondria play a key role in the regulation of apoptosis and ROS production. As the power source of the cell, mitochondria are the main consumers of O_2_ and the main source of ROS [33,43,44]. To further understand the mechanism of DBD plasma-induced *F. oxysporum* inactivation, the mitochondrial potential was monitored by flow cytometry in the current study, and the result is shown in Figure 6. Population 1 (P1) is a negative cell population with red fluorescence, and Population 2 (P2) is a positive cell population with green fluorescence. When the O_2_ content was 20%, the proportion of positive cells was 29.05% after 1 min of the DBD plasma treatment, and it increased by 19.74% after 5 min of the DBD plasma treatment. When the O_2_ content was 40%, the positive cell population was 31.87% after 1 min of the treatment, and the positive cell population was 53.8% when the treatment time was extended to 5 min. It can be seen that increasing the O_2_ content in the working gas and extending the treatment time can aggravate the depolarization of the mitochondrial membrane. The destruction of mitochondria is a hallmark event in the early onset of apoptosis. The depolarization of the mitochondrial membrane potential causes mitochondrial dysfunction, reduces signaling, decreases ATP synthesis, and triggers a cascade reaction of apoptotic enzymes, ultimately leading to spore apoptosis [45]. With the decrease in the mitochondrial membrane potential and the destruction of mitochondria, the content of endogenous ROS should be decreased. However, it can be seen in Figure 4 that the ROS content in the spores increased, indicating that the ROS came from outside the cell, which may be due to the destruction of the cell membrane, making it easier for ROS to enter the spores. Therefore, mitochondrial damage may be caused by the accumulation of exogenous ROS in the spores and its induced oxidative stress.

### 2.7. Effect of DBD Plasma Treatment on Free Fatty Acids

**Figure 7:**
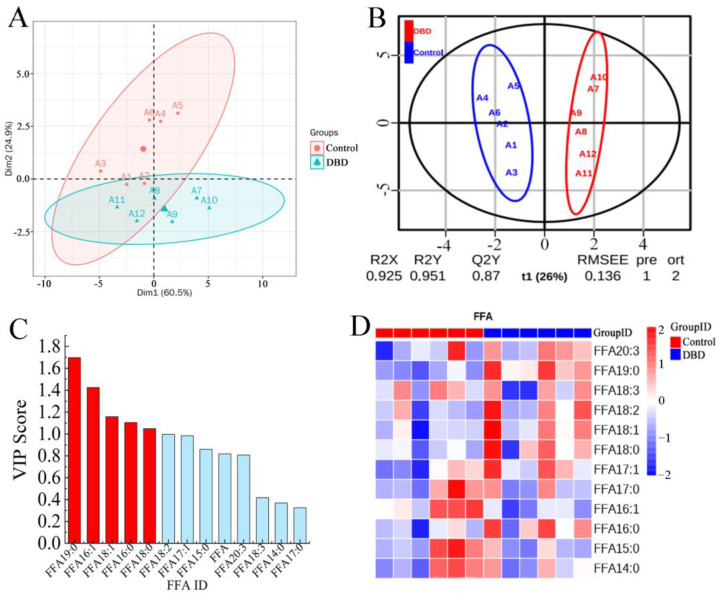
Analysis of free fatty acid metabolism. (**A**) PCA score chart of the control and DBD-treated groups. (**B**) PLS-DA score chart of the control and DBD-treated groups. (**C**) VIP score chart (red columns indicate FFAs with VIP > 1). (**D**) Heat map of metabolic differentials in the control and DBD-treated groups.

Fatty acids are a source of cellular energy and an important component of cell membranes [46]. In this study, a total of 12 free fatty acids were tested. According to the PCA score plot (Figure 7A), there were no significant outliers between the two groups of samples. Moreover, there were significant differences in the first two principal component dimensions between the treatment and control groups, which indicates that the DBD plasma treatment caused changes in the free fatty acid content within the spores. To distinguish potential differences in FFAs between the untreated and DBD plasma-treated groups, the PLS-DA model was used to analyze and predict the obtained data, which are shown in Figure 7B. VIP predict > 1 is an important indicator for differentiating between differential free fatty acids (Figure 7C). The research data showed that FFA19:0, FFA16:1, FFA18:1, FFA16:0, and FFA18:0 could be used as important indicators to distinguish the two groups. Heat map are also often used to observe the overall difference of the sample, where colors closer to red mean a higher metabolite content and colors closer to blue mean a lower metabolite content. In Figure 7D, we can see that there is a significant difference in the FFAs between the untreated group and the DBD plasma-treated group. All in all, the present results support the obvious differential expression of FFAs after the DBD treatment.

**Figure 8:**
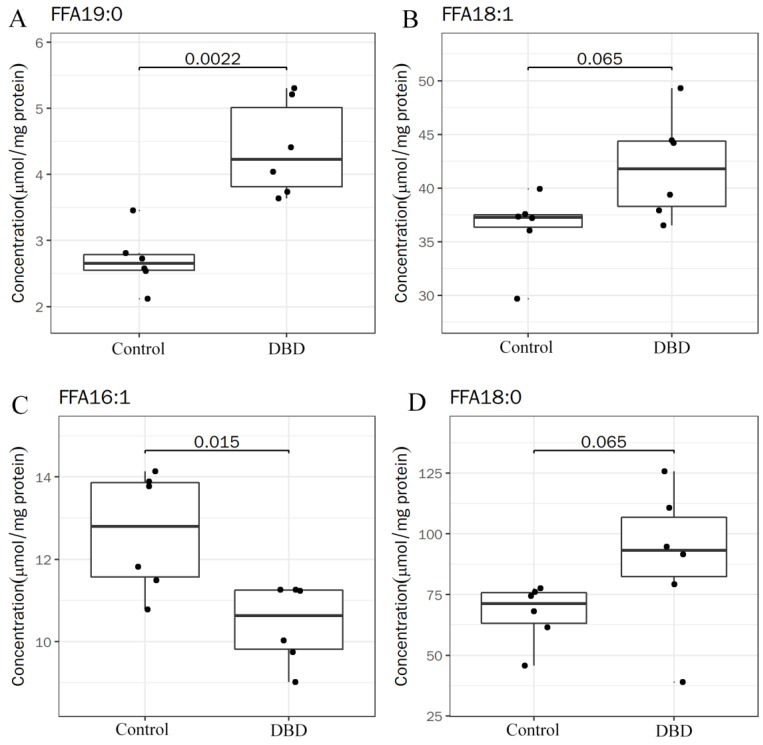
Box plot of differential metabolites: FFA19:0 (**A**), FFA18:0 (**B**), FFA18:1 (**C**), and FFA16:1 (**D**).

Figure 8 shows the four lipids with the most significant differences between the untreated and DBD plasma treatment groups (FFA19:0, FFA18:0, FFA18:1, and FFA16:1). where FFA19:0 is the significantly up-regulated lipid and FFA16:1 is the significantly down-regulated lipid. According to the obtained data, the content of saturated fatty acids (FFA19:0, FFA18:0, and FFA16:0) was increased, while the content of unsaturated fatty acids (FFA16:1) was significantly decreased after the DBD plasma treatment compared to the untreated samples. Possibly due to the reaction of plasma-induced ROS with fatty acids, the polyunsaturated fatty acids (PUFAs) near the cell surface are sensitive to ROS and easily oxidized and eventually produce MDA, resulting in an increased MDA content (Figure 5A) [25,26,27,47]. In addition, PUFAs are related to the fluidity of cell membranes. The reduction in UFAs reduces the fluidity of the cell membrane and the ability of the cell membrane to carry out important activities, which will eventually lead to apoptosis. At the same time, the decreased fluidity of the cell membrane also causes the regulation of the osmotic pressure inside and outside the cell, resulting in cell damage or even death due to swelling and rupture. Therefore, we suggest that DBD plasma-induced changes in the fatty acid contents of the cell membrane are one of the important reasons for the inactivation of *F. oxysporum*.

## 3. Materials and Methods

### 3.1. Reagents

Thiobarbituric acid was bought from Shanghai Maclin Biochemical Technology Co., Ltd. (Shanghai, China). 2′,7′-dichlorodihydrofluorescein (DCFH-DA) was provided by Wuhan Science and Technology Co., Ltd. (Wuhan, China). The mitochondrial membrane potential detection kit (JC-1) was purchased from Beijing Biolaibo Technology Co., Ltd. (Beijing, China). The superoxide dismutase (SOD) assay kit, catalase (CAT) assay kit, and reduced glutathione (GSH) assay kit were purchased from the Nanjing Jianjieng Bioengineering Institute (Nanjing, China).

### 3.2. Preparation of Spore Suspensions

*F. oxysporum* was isolated from mango juice in this study, and spore suspensions were made according to Ren et al. [4]. Sterile water (1 mL) was added to the slant medium of *F. oxysporum* to elute *F. oxysporum* for the preparation of the bacterial suspension. The bacterial suspension (200 μL) was added to the PDA medium and coated using a glass spreading rod. The medium containing *F. oxysporum* was placed in constant temperature incubation at 28 °C for 7 days. Subsequently, a phosphate buffer solution (PBS) (0.01 M, pH 7.2, 10 mL) was added to the plate to elute the colonies of *F. oxysporum*. The bacterial suspension was transferred to a conical flask after filtering through four layers of gauze. Finally, the concentration of the bacterial suspension was adjusted to 10^6^ spores/mL as a spare using the hemocyte counting plate.

### 3.3. DBD Plasma Equipment and Treatment Conditions

**Figure 9:**
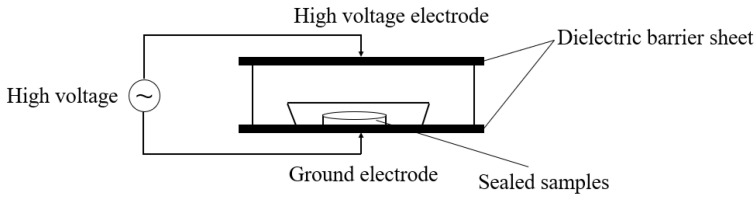
Schematic diagram of the dielectric barrier discharge plasma.

The plasma system used in this study was described in detail in Yi et al. [48]. The DBD plasma device diagram is shown in Figure 9. Sterile Petri dishes containing spore suspensions (10 mL) were placed in rigid polypropylene trays. After exhausting the air, they were filled with working gas and sealed with a polyamide/polyethylene composite film in a MAP-H360 packaging machine, and then treated with the BK-130 DBD cold plasma system (75 kV). The treatment conditions used in this study were slightly modified according to previous studies [49,50]. A mixture of O_2_ (99.2%) and CO_2_ (99.9%) was used as the gas source; the proportion of O_2_ in the mixture was changed to 20%, 30%, and 40%, and then, the sample was treated with DBD plasma for 1, 3, and 5 min.

### 3.4. Bactericidal Effect of DBD Plasma on Fusarium Acuminatum

The spore suspensions (10 mL) were treated with the DBD plasma system with different O_2_ levels (20%, 30%, and 40%) for different time durations (1, 2, 3, 4, and 5 min). The treated spore suspension was serially diluted 10-fold in PBS, and subsequently, the diluted solution (100 µL) was spread on a PDA medium. After 5 days of incubation at 28 °C, the number of colonies was counted, and the results are expressed as log CFU/mL.

### 3.5. Field Emission Transmission Electron Microscope (FETEM)

An FETEM (Thermoscientific Talos F200X G2, Redmond, WA, USA) was used to observe the changes in the spore morphology before and after the DBD plasma treatment with reference to a previous description [12]. The spore suspension (10 mL) was taken out and treated with DBD plasma at 75 kV and 40% O_2_ for 3 min and 5 min, respectively. Finally, the spore morphology was observed using the FETEM, while those not treated with DBD plasma were set as the control group.

### 3.6. Effects of DBD Plasma on Cell Membrane Integrity

The effect of DBD plasma on the cell membrane integrity was assessed by measuring the relative conductivity and protein and nucleic acid leakage. The quantity of nucleic acid and protein leakage within the spores was determined with a UV spectrophotometer [51]. Firstly, the spore suspension was centrifuged (8000× *g*, 4 °C) to obtain the supernatant after the DBD plasma treatment. The absorbance values of the supernatant were determined at 260 nm and 280 nm, where the nucleic acid leakage was expressed as the absorbance value at 260 nm, while the protein leakage was expressed as the absorbance value at 280 nm.

The spore suspension (4 mL) was centrifuged to collect the precipitate at 8000× *g* for 5 min at 4 °C. The relative conductivity was determined after precipitation and incubation with PBS at 25 °C for 15 min. The conductivity was determined again after boiling for 15 min, and then, the relative conductivity was calculated according to the following formula:

Relative conductivity (%) = (A_2_ − A_1_)/A_1_ × 100% (1)

where A_1_ is the conductivity measured after incubation at 25 °C for 15 min and A_2_ is the conductivity measured after boiling for 15 min.

### 3.7. Determination of ROS Level

The fluorescent probe 2′,7′-dichlorodihydrofluorescein (DCFH-DA) was used to detect the levels of intracellular ROS in this study. Simply, the DBD plasma treatment was applied to treat the spore suspensions for 1, 3, and 5 min at 75 kV with 20%, 30%, and 40% O_2_, respectively. Then, the treated spores were collected and washed with PBS before adding the DCFH-DA probe. Subsequently, the spores were washed 3 times with PBS to remove the DCFH-DA that did not sufficiently enter the cells after being incubated in the dark at 37 °C for 20 min. The content of ROS was quantified by flow cytometry (CytoFLEX, Brea, CA, USA) within 30 min, as previously described [52].

### 3.8. Lipid Peroxidation Assay

Malondialdehyde (MDA), as a degradation product of lipid peroxidation, is often used to reflect the degree of lipid peroxidation, which indirectly measures the degree of cellular damage. In this study, we used thiobarbituric acid to detect the MDA content [53], due to the fact that the condensation of malondialdehyde with thiobarbituric acid produces a red product. Simply, the prepared spore suspension (10 mL) was taken and treated with DBD plasma at 75 kV, with different O_2_ contents (20%, 30%, and 40%) and for different time durations (1, 3, and 5 min), respectively. The treated spore suspension (4 mL) was centrifuged at 8000× *g* for 5 min at 4 °C, and then, the supernatant was mixed with thiobarbituric acid (0.67%, 2 mL) and reacted in a boiling water bath for 15 min. Finally, the absorbance values were measured at 450 nm, 532 nm, and 600 nm, using a UV spectrophotometer (TU1810, Beijing Purkinje General Instrument Co., Ltd., Beijing, China). The MDA content was calculated according to the following equation:

MDA concentration (μmol/L) =6.45 × (A_532_ − A_600_) − 0.56A_450_ (2)

### 3.9. Determination of SOD and CAT Activity and GSH Content

First, the spore suspensions (10 mL) were treated by DBD plasma for different time durations (1, 3, and 5 min) at different O_2_ levels (20%, 30%, and 40%), respectively. Subsequently, the treated spore suspension (4 mL) was centrifuged at 8000× *g* for 5 min at 4 °C to collect spores and then resuspended in PBS. Intracellular enzymes were released through ultrasound (JYD-900L, Shanghai, China) under the conditions of (power 300 W, 5 s intervals, and 10 min). Finally, enzyme activity assays were performed using the SOD, CAT, and GSH assay kits (Nanjing Construction Biology, Institute, Nanjing, China).

### 3.10. Detection of Mitochondrial Membrane Potential

When the membrane potential is normal, JC-1 enters the mitochondria through the mitochondrial membrane and the concentration rises to form a multimer that emits red fluorescence. In apoptotic cells, the mitochondrial membrane is depolarized, and JC-1 is released from the mitochondria at a reduced concentration to form green fluorescent monomers [52]. Therefore, we usually use the red-to-green fluorescence ratio to reflect the degree of mitochondrial membrane depolarization. Spore suspensions (10 mL) were treated with DBD plasma for different time durations (1, 3, and 5 min) at different O_2_ levels (20%, 30%, and 40%), respectively. Subsequently, staining was performed according to the JC-1 fluorescent probe instructions and then analyzed by flow cytometry (CytoFLEX, Brea, CA, USA).

### 3.11. Metabolism of Free Fatty Acids

The spore suspension (10 mL) was treated with DBD plasma at 75 kV with 40% O_2_ for 5 min, and the non-DBD treated spore suspension was used as the control. The FFAs were extracted from fungal spores according to Lam et al. [54]. First, the spores were homogenized in 750 µL of a chloroform: methanol 1:2 (*v*/*v*) solution containing 10% deionized water and incubated at 4 °C for 30 min before adding deionized water (350 µL) and chloroform (250 µL). After centrifugation, the lower organic phase containing lipids was extracted twice and the extract was collected. Then, the samples were dried and stored at −80 °C. Finally, the samples were analyzed for fatty acids using Shimadzu Nexera 20AD-HPLC and a triple quadrupole/ion trap mass spectrometer (6500 Plus QTRAP; SCIEX, Framingham, MA, USA) according to Lam [55].

### 3.12. Statistical Analysis

All experiments were conducted in triplicate and calculated as the mean ± standard deviation (SD). Particularly, the determination of the FFA content was repeated six times. Statistical significance and a one-way analysis of variance were performed with the SPSS 26.0 statistical program (IBM Co., Somers, NY, USA). *p* < 0.05 was considered a significant difference. Graphs were drawn using Origin 2024 (Origin Lab Co., Northampton, MA, USA).

## 4. Conclusions

Exploring the mechanism of action of DBD plasma on the inhibition of *F. oxysporum* is helpful to provide a new idea for the sterilization of *F. oxysporum*. In this study, the data support that DBD plasma has a good sterilization effect on *F. oxysporum*. In addition, extending the treatment time and increasing the proportion of O_2_ in the gas mixture can improve the sterilization efficiency. The results of lipid peroxidation and free fatty acid metabolism show that ROS produced by DBD plasma leads to the peroxidation of membrane lipids, causing serious damage to the cell membrane and resulting in the leakage of intracellular components. Through the determination of intracellular ROS levels and ROS-related components, we concluded that the ROS produced by DBD plasma induced oxidative stress in cells, with a significant increase in intracellular ROS levels and a decrease in related enzyme activities. Moreover, we found that the mitochondria were seriously damaged. In conclusion, oxidative stress induced by ROS produced by DBD plasma is an important cause of cell death.

Taken together, these results provide new insights into the mechanisms by which DBD plasma inhibits *F. oxysporum* and support DBD plasma as a promising alternative to eliminate *F. oxysporum* from food production. However, due to the characteristics of the DBD plasma equipment, the number of samples processed at each time is limited; so, whether DBD plasma sterilization can be applied to the food industry on a large scale remains to be considered. In addition, the bactericidal effect of DBD plasma on *F. oxysporum* in different forms of food also needs to be further studied.

## Data Availability

Data will be made available upon request.
